# Kawasaki Disease Diagnosis and Treatment in over 1000 Patients: A Continuum of Dysregulated Inflammatory Responses

**DOI:** 10.3390/biomedicines12092014

**Published:** 2024-09-03

**Authors:** Stejara A. Netea, Giske Biesbroek, Diana van Stijn, Sietse Q. Nagelkerke, Kawasaki Study Group, CAHAL Group, KIRI Group, Irene M. Kuipers, Taco W. Kuijpers

**Affiliations:** 1 Pediatric Immunology, Rheumatology and Infectious Disease, Emma Children’s Hospital, Amsterdam UMC, Meibergdreef 9, 1105 AZ Amsterdam, The Netherlands; g.biesbroek@amsterdamumc.nl (G.B.); d.vanstijn@amsterdamumc.nl (D.v.S.); s.q.nagelkerke@amsterdamumc.nl (S.Q.N.); t.w.kuijpers@amsterdamumc.nl (T.W.K.); 2 Department of Experimental Immunology, Amsterdam Institute for Infection & Immunity, Amsterdam UMC, University of Amsterdam, 1105 AZ Amsterdam, The Netherlands; 3 Department of Molecular Hematology, Sanquin Research Institute, Plesmanlaan 125, 1066 CX Amsterdam, The Netherlands; 4 Pediatric Cardiology, Emma Children’s Hospital, Amsterdam UMC, Meibergdreef 9, 1105 AZ Amsterdam, The Netherlands; i.m.kuipers@amsterdamumc.nl

**Keywords:** Kawasaki disease, KD, mucocutaneous lymph node syndrome, MIS-C, multisystem inflammatory syndrome in children, hyperinflammation, epidemiology, phenotypic patterns

## Abstract

Background: Kawasaki disease (KD) is a pediatric vasculitis, leading to coronary artery aneurysms (CAAs) in ~4–14%. Attention to the etiology and course of KD was generated by the close mimic of a SARS-CoV-2-induced phenotype, called multisystem inflammatory syndrome in children (MIS-C). Methods: A total of 1179 cases were collected from 2012 with ~50% of cases retrospectively included. Clinical characteristics were described and risk factors for CAA (persistence) were investigated. Phenotypic patterns of the prospectively included KD patients were evaluated. These patterns were also compared to the seronegative KD and seropositive MIS-C cases identified during the SARS-CoV-2 pandemic. Results: KD mostly affected boys and children < 5 years. IVIG resistance, CAAs, and giant CAAs occurred in 24.5%, 21.4%, and 6.6%, respectively. Giant CAAs were significantly more likely to normalize to a normal *Z* score in patients that were younger than 2.5 years old at the time of initial giant CAA (χ^2^ test *p* = 0.02). In our prospective (SARS-CoV-2-seronegative) KD series, there was a diminishing male predominance over time, whereas the proportions of incomplete presentations (*p* < 0.001) and patients with circulatory shock (*p* = 0.04) increased since the COVID-19 pandemic. Pre- and post-pandemic KD cases presented with different levels of C-reactive protein, thrombocyte counts, and hemoglobin levels over the years. Compared to pandemic KD, SARS-CoV-2-seropositive MIS-C patients were older (*p* < 0.001), and more often required intensive care admission (*p* < 0.001), with a gradual decrease over time between 2020 and 2022 (*p* = 0.04). KD carried a substantial risk of CAA development in contrast to MIS-C. Conclusion: the phenotypic changes seen over the last twelve years of our prospective follow-up study suggest a spectrum of hyperinflammatory states with potentially different triggering events within this clinical entity.

## 1. Introduction

Kawasaki disease (KD) is a severe pediatric vasculitis, which was first described in Japan over five decades ago [1]. It was hypothesized to be a post-infectious hyperinflammatory syndrome in genetically susceptible children, but the exact mechanism behind Kawasaki disease remains unknown [2,3]. KD has a male preponderance and most often affects children under the age of five [4,5]. The most important complication of KD is the development of coronary artery aneurysms (CAAs), which develop in about 15–25% of untreated patients and can lead to thrombus formation or stenosis, with an increased risk of myocardial infarction [6,7]. Although this risk significantly diminished since the introduction of standard treatment with intravenous immunoglobulins (IVIG) and aspirin [8], IVIG resistance occurs in up to 20% of affected children [8] and 4–14% of children develop CAAs despite timely treatment, stressing the need to elucidate mechanisms underlying IVIG resistance and CAA development. 

In this context, the Dutch Kawasaki Disease Study was established in 2012 to improve the diagnosis, follow-up, and treatment of KD by investigating its etiology, presentation, and long-term consequences leading to a total number of over 1000 patients. As a tertiary referral center from 1999 onwards, most patients are from the larger Amsterdam region, which serves about 5 million inhabitants in total. Because of its overlapping clinical and immunological features, patients with the severe SARS-CoV-2-related multisystem inflammatory syndrome in children (MIS-C) were included in this study as well [2,3]. 

The current manuscript focuses on the clinical presentation of patients with KD before and after the COVID-19 pandemic, describing the longitudinal phenotypic patterns observed in our cohort and comparing KD to SARS-CoV-2-seropositive KD/MIS-C cases during the COVID-19 pandemic. While the overall clinical phenotype of KD was consistent with its presentation described in other studies, a high proportion of CAAs (21.4%) was found with a slighter chance of normalization of giant CAAs in children above 2.5 years old at the time of the initial CAA. Additionally, several phenotypical changes were observed. There was a diminishing male predominance over time, more incomplete presentations, more patients with circulatory shock and varying over time levels of C-reactive protein, thrombocyte counts, and hemoglobin levels. MIS-C patients were older and more often required intensive care unit (ICU) admission, although the number of patients requiring ICU admission gradually decreased over the years of the pandemic. 

## 2. Materials and Methods

### 2.1. Study Design and Population

**Figure 1:**
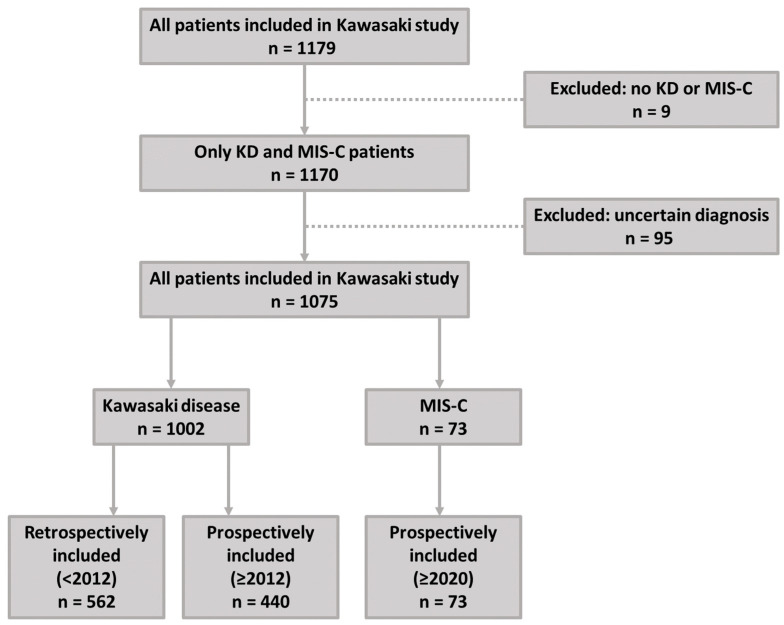
Inclusion flowchart.

Patients between 0 and 18 years old with KD [8,9] or MIS-C [10] between June 1976 and January 2024 were included at the Amsterdam UMC, and participating hospitals in the broad Amsterdam region (Noordwest Hospitalgroup Alkmaar, Amstelland, Flevohospital, Haga Hospital, Catharina Hospital, Dijklander Hospital, Leiden University Medical Center, Maasstad Hospital, Meander, Onze Lieve Vrouwe Gasthuis, Reinier de Graaf Group, Spaarne Hospital, and Saint Antonius Hospital, Tergooi). Data from patients diagnosed with KD between 1976 and 2012 were collected retrospectively, while patients identified with KD or MIS-C in or after the study initiation in 2012 were included prospectively (Figure 1). KD patients enrolled after the emergence of COVID-19 were proven as SARS-CoV-2 seronegative by lack of antibodies against the spike antigen or nucleocapsid protein until April 2022.

### 2.2. Data Collection

Clinical data from the acute disease episode and consequent appointments at the outpatient clinic were collected and combined in an electronic health database (Castor) under a study ID. Information regarding clinical presentation (i.e., age, sex, first day of illness, symptoms, laboratory outcomes [nadir laboratory value within first 2 weeks post-onset], treatment, complications, and cardiovascular manifestations) were collected. As per the American Heart Association guidelines, primary treatment consisted of a single IVIG infusion (2 g/kg) and aspirin (started 30–50 mg/kg/day and reduced to 3–5 mg/kg/day once fever subsided for 48 h) [8,9]. IVIG resistance was defined as persistent or recurrent fever > 38 degrees Celsius at >36 h and <7 days after completing initial IVIG infusion in patients treated with IVIG ≤10 days post-onset of fever [8]. In case of IVIG resistance, additional treatment with corticosteroids (methylprednisolone pulse, 30 mg/kg/day for 3 days) was initiated, sometimes followed by biologicals (infliximab, anakinra). Cardiac imaging was performed in the acute and convalescent phase by echocardiography to evaluate the cardiac function and presence of CAAs by Z score. CAAs were defined as a Z score ≥ 2.5 (small ≥ 2.5–5.0, medium ≥ 5.0–10, and giant ≥ 10) [8]. Importantly, echocardiography measurements for coronary artery assessment improved over time, especially since introduction of the Vivid 6 in 2007. Currently, our center uses the Vivid E95 cardiac ultrasound system (General Electric Vingmed Ultrasound, Horten, Norway) using EchoPAC software version 206 (revision 66.0). Although not included as part of the analyses in the current study, we also collected plasma EDTA samples from the patients concurrent with clinical blood draws (<2 years old 2 mL, >2 years old 4.5 mL) during admission and at the outpatient clinic. After collection, plasma samples were aliquoted and stored at −20 degrees and not subjected to multiple freeze–thaw cycles.

Clinical data from the acute disease episode and consequent appointments at the outpatient clinic were collected and combined in an electronic health database (Castor) under a study ID. Information regarding clinical presentation (i.e., age, sex, first day of illness, symptoms, laboratory outcomes [nadir laboratory value within first 2 weeks post-onset], treatment, complications, and cardiovascular manifestations) were collected. As per the American Heart Association guidelines, primary treatment consisted of a single IVIG infusion (2 g/kg) and aspirin (started 30–50 mg/kg/day and reduced to 3–5 mg/kg/day once fever subsided for 48 h) [8,9]. IVIG resistance was defined as persistent or recurrent fever > 38 degrees Celsius at >36 h and <7 days after completing initial IVIG infusion in patients treated with IVIG ≤10 days post-onset of fever [8]. In case of IVIG resistance, additional treatment with corticosteroids (methylprednisolone pulse, 30 mg/kg/day for 3 days) was initiated, sometimes followed by biologicals (infliximab, anakinra). Cardiac imaging was performed in the acute and convalescent phase by echocardiography to evaluate the cardiac function and presence of CAAs by Z score. CAAs were defined as a Z score ≥ 2.5 (small ≥ 2.5–5.0, medium ≥ 5.0–10, and giant ≥ 10) [8]. Importantly, echocardiography measurements for coronary artery assessment improved over time, especially since introduction of the Vivid 6 in 2007. Currently, our center uses the Vivid E95 cardiac ultrasound system (General Electric Vingmed Ultrasound, Horten, Norway) using EchoPAC software version 206 (revision 66.0). Although not included as part of the analyses in the current study, we also collected plasma EDTA samples from the patients concurrent with clinical blood draws (<2 years old 2 mL, >2 years old 4.5 mL) during admission and at the outpatient clinic. After collection, plasma samples were aliquoted and stored at −20 degrees and not subjected to multiple freeze–thaw cycles.

### 2.3. Statistics

First, we compared the retrospective vs. prospective KD study cohorts. Categorical clinical characteristics were summarized by frequencies and percentages. Continuous outcomes were evaluated by mean and standard deviation or median and interquartile range for variables with a normal and non-normal distribution, respectively. Differences between retrospective and prospective data were compared (χ^2^, Mann–Whitney U). Since no difference was found in the risk of CAA formation, a multivariable regression analysis was conducted to investigate the association of various clinical factors (i.e., male sex, age at onset <1 year old, IVIG initiation >10 days post-onset of symptoms and IVIG resistance, blood value measurements (i.e., C-reactive protein, leukocytes, thrombocytes, hemoglobin, aspartate transaminase [ASAT], and alanine transaminase [ALAT]) with the development of ([persisting] giant) CAAs in the full cohort. In order to do so, we performed univariate analyses to identify important covariates and selected variables whose *p* values were below 0.25. We then fit the multivariable model by stepwise exclusion of variables based on the Wald statistic and identifying the model with best goodness of fit based on the significance of the model and Nagelkerke R^2^. 

We focused our further longitudinal analyses on the over-time phenotypic changes of the prospectively included patients, since the groups differed slightly in some of the other clinical aspects and the higher reliability of prospective data. First, we described the clinical phenotype of prospectively included KD patients prior to the COVID-19/MIS-C pandemic (<2020), during the pandemic (≥2020 to April 2022), and after the pandemic (>April 2022) (Fisher–Freeman–Halton exact and Kruskal–Wallis). We also conducted annual analyses for prospectively included patients (all patients included between 2012 and 2024), specifically focusing on trends for the most important changes seen in the 4-year timeframes.

We then described the clinical characteristics of MIS-C patients separately and compared these to pandemic KD (χ^2^, Mann–Whitney U). Annual longitudinal changes were investigated for MIS-C as well.

Significance values were adjusted by Bonferroni correction for multiple tests. Statistical analyses were performed in IBM SPSS Statistics 25 and R studio 4.2.1. Differences of *p* < 0.05 were considered statistically significant. Graphs were created in GraphPad Prism 8.4.2.

### 2.4. Study Approval

The Medical Research Ethics Committee of the Amsterdam UMC provided ethical approval for the Kawasaki Disease Study (reference number: 2012_155, NL41023.018.12, 21 June 2012). Approval by the Medical Research Ethics Committee was obtained for all participating centers as well in the respective centers. 

Informed consent and written approval were explicitly obtained for all retrospectively and prospectively included patients. Approval of retrospectively included patients (Kawasaki episode before 2012) was obtained by the Kawasaki study team after referral by the treating physician to the Amsterdam UMC for post-hoc follow-up after initiation of the Kawasaki study. These consisted of patients diagnosed with KD previously or post-hoc because of admittance for cardiac events at an adult age. Approval of the prospectively included patients was obtained in the Amsterdam UMC or in one of the participating centers during the acute disease episode. After initial identification of a patient adhering our inclusion criteria, the patient was informed about the study procedures by a local researcher with Good Clinical Practice and BROK certification. This included a detailed description surrounding collection and pseudo-anonymization of the data, as stated in Section 2.2 (Data Collection section). Informed consent and written approval were obtained in accordance with the study protocol, the International Conference on Harmonization Good clinical Practice guidelines, and the provisions of the Declaration of Helsinki.

## 3. Results

### 3.1. Patient Inclusion

A total of 1179 patients were included in the Kawasaki Disease Study until January 2024 (Figure 1). Of these patients at our center, 1002 KD and 73 MIS-C patients were included in our current report after exclusion of cases misdiagnosed with KD or MIS-C and/or uncertainty concerning the diagnosis. The latter consisted of patients with loss to follow-up and missing data concerning timing of diagnosis, initial presentation, and treatment; patients included after the acute phase when the symptoms resolved and with uncertainty surrounding the initial diagnosis in other hospitals; or patients with CAAs identified in adulthood and a possible, but non-documented, KD episode in the past. Of the 1002 KD patients, 562 were included retrospectively during follow-up (with a data-of-onset prior to start of Kawasaki Disease Study in 2012), and 440 were included prospectively from 2012 onwards. All MIS-C patients were included prospectively.

### 3.2. Kawasaki Disease

#### 3.2.1. Clinical Phenotype of Retrospective and Prospective KD Study Population

The baseline and clinical characteristics of our complete KD cohorts included prior to 2012 (retrospectively) and from 2012 onwards (prospectively) are displayed in Table 1. KD patients enrolled after the emergence of COVID-19 during the MIS-C pandemic were proven SARS-CoV-2 seronegative (between January 2020 and April 2022). 

KD patients included more boys than girls (ratio 3:2). Most of the children were younger than 5 years at the time of diagnosis (78.2%), with a slightly younger age at onset in the retrospectively included patients (2.3 vs. 3.1 years old, *p* < 0.001). A majority were of full Dutch origin with four grandparents born in The Netherlands (59.6%). Patients of non-Dutch ancestry were mostly from Suriname, Morocco, or Turkey. Most children were diagnosed in winter. A majority of children had a complete disease presentation (78.8%), presenting with polymorphic exanthema, conjunctivitis, cervical lymphadenopathy, changes in the mucosa and/or changes in the extremities in about 80–90% of the cases. Changes in the extremities were significantly more often reported by the patients included retrospectively (83.4% vs. 77.8%, *p* = 0.04), resulting in a complete KD diagnosis more often (81.3% vs. 75.5%, *p* = 0.03). Rare KD manifestations included non-coronary aneurysms, papilledema and uveitis, (bilateral) sensorineural hearing loss, concomitant autoimmune neutropenia or immune thrombocytopenia, and familial hypercholesterolemia (as tested during follow-up), which were seen in <1% of all cases.

A majority of the patients were treated according to standard protocol with IVIG (n = 912 [91.5%]) at a single high dose of 2 g/kg (n = 678 [75.5% of total population]) and within 10 days of onset of fever (n = 711 [80.7% of total population]). In 85 patients (8.5%) IVIG was not administered due to late or retrospective KD diagnosis in cases that no longer had fever. Treatment with a second IVIG infusion (20.9% vs. 28.9%, *p* = 0.03) or corticosteroids (5.8% vs. 14.8%, *p* < 0.001) was reported less often for retrospectively included patients. Biologics were initiated in seven (0.7%) patients, with five patients receiving anakinra and two receiving infliximab. Importantly, we emphasize that our treatment protocol never changed the standard treatment of a single high-dose IVIG infusion, followed by a second dose if IVIG resistance occurred, before adding additional disease-modifying drugssuch as corticosteroids or biologicals to treat KD.

CAAs developed in 21.4% of the patients with no significant difference between the development of CAAs in the retrospective vs. prospective cohorts. Of these, 72.4% were boys, 40% were <1 year old at presentation, 34.0% did not receive IVIG treatment ≤ 10 days of onset of symptoms, and 36.6% were IVIG resistant. In a multivariable analysis, male sex, age < 1 year at presentation, treatment > 10 days after onset of symptoms, and IVIG resistance were independent risk factors for the development of CAAs (Table 2A). Similarly, male sex, age < 1 year at presentation, treatment > 10 days after onset of symptoms, IVIG resistance, and CRP > 118.0 mg/L were independent risk factors for giant CAAs (Table 2B).

Of the 95 patients with small CAAs, only 2.3% (n = 2) persisted as small CAAs. Of the 43 patients with medium CAAs, only 11.9% (n = 5) persisted as a small (50.0% [n = 2]) or medium (50.0% [n = 2]) CAA. Of the 61 patients with giant CAAs (6.6%), 32.2% (n = 19) normalized during follow-up and 67.8% (n = 40) had persisting CAAs. Of the latter, 42.4% (n = 8) persisted as giant CAAs, 33.3% (n = 11) regressed to a medium CAA, and 24.2% (n = 14) regressed to a small CAA. Patients with giant CAAs were significantly more likely to normalize to a normal *Z* when younger than 2.5 years old at the time of initial presentation with giant CAA (χ^2^ test *p* = 0.02). This association remained significant (*p* = 0.04, odds ratio 0.2 [95% confidence interval 0.06–0.96]) in a multivariable logistic regression including treatment >10 days of onset of symptoms, which was not associated with persisting CAAs.

In conclusion, while the overall KD phenotype in our retrospective and prospective cohorts both adhered to the phenotype described previously by other groups, a large proportion of children developed CAAs, which can be explained by the fact that the Amsterdam UMC is a tertiary referral and expertise center for KD. In the most severe patients with giant CAAs, we observed that the *Z* scores were more likely to normalize in children younger than 2.5 years old at onset, which is highly relevant for anticoagulation therapy and follow-up monitoring. 

#### 3.2.2. Clinical Phenotype of Pre-Pandemic, Pandemic, and Post-Pandemic KD

Some clinical parameters differed between the retrospective vs. prospective KD patients, because for the patients that developed KD < 2012, we depended on documentation and former echocardiography machines available at the time of the disease episode, and sometimes the recollection of the treating physician, patients, and/or parents. Since these data are prone to technical differences in resolution, recall, and selection bias, we excluded the retrospectively included patients from further (longitudinal) analyses. 

**Figure 2:**
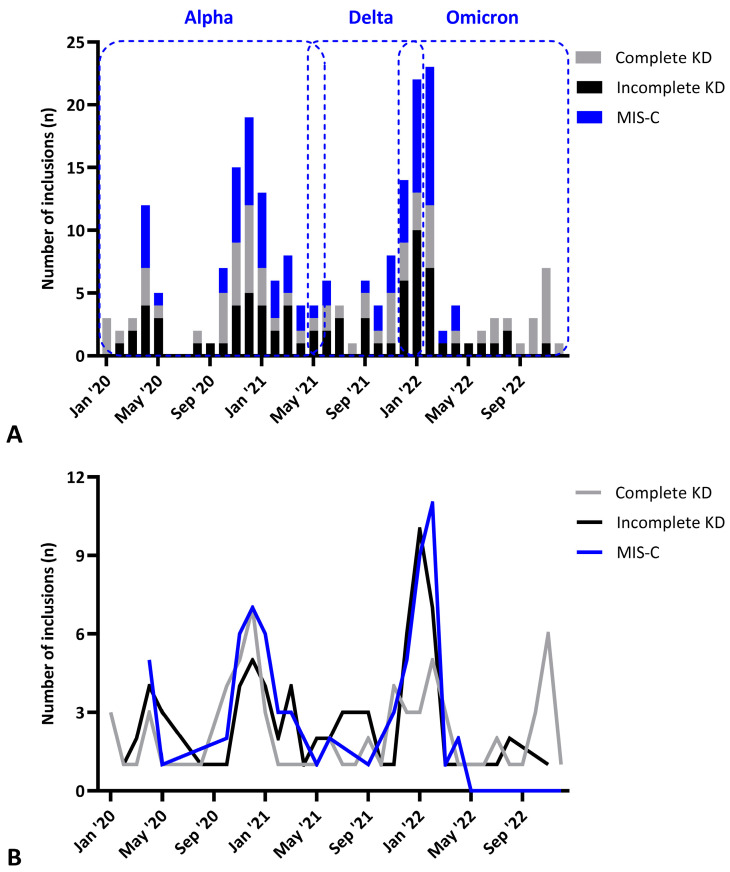
Number of complete KD, incomplete KD and MIS-C inclusions in the Amsterdam cohort in the COVID-19 pandemic with the dominating strains of SARS-CoV-2 depicted in the dashed frames shown per month of inclusion (**A**) and continuously over-time (**B**).

Using the prospectively collected dataset only, the longitudinal incidence, seasonality, and phenotypic changes of prospectively included KD were investigated before and after initiation of the COVID-19 (and MIS-C) pandemic. The incidence of KD since the start of the COVID-19 pandemic decreased from a total of 109 KD patients in 2017–2019 to an 83 SARS-CoV-2-seronegative KD patients in an equal period in 2020–≤2022. Interestingly, the SARS-CoV-2-negative KD cases diagnosed during the pandemic mimicked the patterns of the MIS-C inclusion peaks (Figure 2), being most notable for the incomplete KD cases.

The total group of prospectively included patients (date of onset ≥ 2012) consisted of 440 KD patients, being 329 pre-pandemic, 60 SARS-CoV-2 seronegative pandemic (2020 to April 2022), and 51 post-pandemic KD patients (after April 2022 [after which no more MIS-C patients were identified in The Netherlands]). Baseline and clinical characteristics of these KD cohorts are displayed in Table 3.

**Figure 3:**
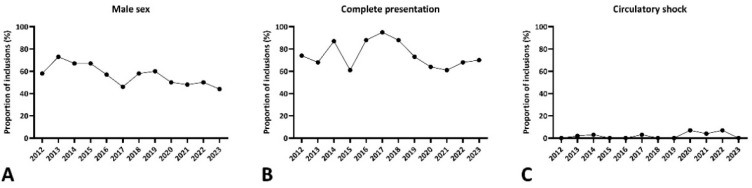
Annual trends seen for male sex distribution (**A**), complete presentations (**B**) and circulatory shock (**C**) in prospectively included patients (all patients included between 2012 and 2023).

While there was a clear male predominance in KD within the timeframe of 2012 to <2016 (66.3%), this was no longer the case in the later timeframes with a 4:5 ratio between 2016 and <2020 and a 1:1 ratio after 2020 (*p* = 0.02). Consistently, we found a general annual trend towards a decreased male predisposition (Figure 3A). Additionally, significantly more KD cases presented with an incomplete presentation in the pandemic and post-pandemic cohorts compared to the timeframes between 2012 to <2016 (13.8%) and 2016 to <2020 (28.1%) (*p* < 0.001), consistent with the lower proportions of patients presenting with cervical lymphadenopathy (*p* = 0.04) and changes in the extremities (*p* = 0.005). This distribution between complete vs. incomplete presentations varied annually as well, with no clear trend observed (Figure 3B). Additionally, more SARS-CoV-2-seronegative KD patients presented with circulatory shock during and after the MIS-C pandemic (4.5%) than before (*p* = 0.02), a trend that was also observed in our annual analyses (Figure 3C).

Regarding the blood measures, CRP values were significantly higher between 2016 and <2020 compared to the 4-year timeframe before (*p* = 0.003); thrombocyte levels were significantly higher between 2022 and ≤2024 (post-pandemic) compared to 2012 to <2016 (*p* = 0.05); hemoglobin levels were significantly lower between 2020 and <2022 (pandemic, *p* < 0.001) and 2022 to <2024 (post-pandemic, *p* = 0.005) compared to 2012 to <2016.

No significant changes were seen in the proportion of children treated with IVIG within 10 days of the onset of symptoms, the proportions of patients admitted to the ICU, or in the proportions of patients presenting with small, medium, or giant CAAs within the first six weeks of presentation, although giant CAAs were observed less often (not significant). 

No annual changes in KD phenotype were seen since the initiation of the pandemic, apart from the median higher age of KD patients between 2020 (4.3 [IQR 2.1–8.3] years) and 2023 (2.5 [IQR 0.7–3.9] years) (*p* = 0.04) (Table 4). 

In summary, various changes were noticed in KD phenotype before, during, and after the COVID-19 and MIS-C pandemic.

### 3.3. Multisystem Inflammatory Syndrome in Children during the SARS-CoV-2 Pandemic

#### 3.3.1. Clinical Phenotype of MIS-C Compared to KD during the MIS-C Pandemic

Between April 2020 and April 2022, 73 MIS-C patients were prospectively included in our study, after which no more MIS-C patients presented in The Netherlands (Table 5). The clinical characteristics of MIS-C patients overlapped with a classic/complete presentation of KD in 31.0% of cases. The MIS-C cases were defined based on SARS-CoV-2-seropositivity against the spike antigen plus a current or recent SARS-CoV-2 infection based on RT-PCR, an antigen test, or a recent COVID-19 infection within the family or a close contact. 

As expected, compared to SARS-CoV-2 seronegative pandemic KD patients, MIS-C patients were significantly older (*p* < 0.001), they presented with the characteristic KD symptoms less often (*p* < 0.001), and had more pronounced gastrointestinal complaints of vomiting or diarrhea (94.3%), which are less prominent in classic KD. MIS-C patients had higher CRP values (*p* < 0.001), and patients more often received treatment with IVIG in <10 days (95.4% vs. 79.6%, *p* = 0.02) and additional treatment with corticosteroids (75.3% vs. 23.7%, *p* < 0.001). Patients more often presented with severe complications, including cardiac dysfunction (*p* < 0.001), respiratory failure (*p* < 0.001), and acute kidney dysfunction (*p* < 0.001), and were consequently admitted to the ICU more frequently (38.4% vs. 5.1%, *p* < 0.001). The same differences were observed when comparing MIS-C patients to the pre-pandemic KD cohort.

Transient CAAs were seen in 9.7% of the MIS-C patients, which was significantly less than in the KD patients (*p* = 0.01). Of these, six patients had a small CAA with Z scores between ≥2.5 and 5.0 (8.3%) and 1 patient had a medium-sized CAA with a Z score between ≥5.0 and 10.0 (1.4%). All normalized within a median of 8 (range 2–193) days post-onset of disease. None of the patients developed giant CAAs (Z scores ≥ 10.0).

#### 3.3.2. Longitudinal Changes of the Clinical Phenotype of MIS-C

The clinical characteristics of MIS-C patients per year of diagnosis did not show any significant change in age at diagnosis, sex distribution, presentation with KD-like characteristics, abdominal symptoms, or CAA formation during these years (Table 6). However, the proportion of MIS-C patients admitted to the ICU significantly fell from 2020 (52.4%) to 2021 (44.8%) and 2022 (17.4%) (*p* = 0.04), in concordance with the drop in patients treated with milrinone (*p* = 0.006) and noradrenaline (*p* = 0.002). Similarly, less patients presented with respiratory failure (*p* = 0.01) and acute kidney injury (*p* = 0.02).

## 4. Discussion

In the current study, we describe the clinical phenotype and longitudinal phenotypic changes of KD (and MIS-C) patients included in the Amsterdam Kawasaki Disease Study over the course of more than two decades. Several phenotypic changes were observed for both pre-pandemic, pandemic, and post-pandemic KD. 

First, although the overall clinical phenotype of KD was consistent with previous studies [4,5,6,7,8,11,12], a relatively high percentage of patients in our cohort developed coronary aneurysms (21.4%), of which 30% were giant CAAs. While this is likely attributed to selection bias by referral to a tertiary expertise center, in our previous 5-year national surveillance study in the whole of The Netherlands, the proportion of patients with CAAs in the Dutch patient population was also at the higher end [12]. In this national study between 2008 and 2012 (n = 341 cases), the mean incidence of KD was estimated to be 5.8/100,000 children < 5 years of age, the median age at disease onset was 2.4 years, and the male-to-female ratio was 1.5 to 1. Incomplete KD was diagnosed in 22.3% of cases and CAAs developed in 13.5%. In line with this study [12] and other previous cohorts, male sex, age < 1 year at presentation, treatment > 10 days after onset of symptoms, as well as clinical IVIG resistance were independent risk factors for the development of CAAs in our cohort, and giant CAAs in particular [13,14]. As expected, giant CAAs were least likely to regress over time [15,16]. Notably, when we stratified these patients by age, we found that giant CAAs were significantly more likely to normalize to a normal *Z* score in patients that were younger than 2.5 years old at the time of initial giant CAAs, remaining significant in a multivariable analysis including male sex and treatment >10 days of onset of symptoms. Whilst we were limited in the number of additional covariates in the model due to the sample size, a previous study (n = 2024) also indicated that older age ≥ 60 months is significantly associated with worse CAA outcomes in terms of improvement vs. progression [17]. It would be interesting to investigate if younger age is indeed a beneficial prognostic factor for CAA regression in other studies. If this is indeed the case, more rigorous treatment may be required in patients with an older age at onset to reduce the incidence of CAAs. It would also be interesting to further investigate if patients at risk for (persisting) CAAs may benefit from adjunctive primary therapy, for instance, with infliximab, as was suggested by the recent trial showing that infliximab is safe, effective, and results in a shorter fever and hospital duration and reduced need for additional therapy [18]. Additionally, yet unidentified predisposing factors may also play a role in the risk of developing giant CAAs, despite timely and adequate treatment.

Second, several longitudinal phenotypical changes were observed within our KD cohort. In line with previous studies, the incidence of KD slightly decreased since 2020, likely due to social distancing during the COVID-19 pandemic [19]. In contrast to the complete phenotype, the incomplete SARS-CoV-2 seronegative KD cases diagnosed during the pandemic mimicked the patterns of the MIS-C inclusion peaks, which seemed to be more related to the winter months. The complete KD cases presented without a clear seasonality essentially similar to the previous time periods, as we also showed in our previous national study [12] (Figure 2). Since KD patients enrolled after the emergence of COVID-19 was proven SARS-CoV-2 seronegative (both RBD seronegative as well as negative for anti-nucleocapsid antibodies until April 2022), misclassification of MIS-C as KD patients is highly unlikely, indicating that the “KD phenotype” may indeed vary over the years.

When separating the prospectively included pre-pandemic KD cases into two equal strata, we observed significant differences in their characteristics with variations in clinical phenotype observed in our longitudinal analyses (e.g., diminishing male predominance over time, more incomplete presentations, and varying levels of C-reactive protein). In line with this, the pre-pandemic and post-pandemic SARS-CoV-2 seronegative KD patients also differed, with (post-) pandemic KD patients having a slightly older age at onset, less male predominance, more incomplete disease presentations, and requiring treatment with additional therapy more often (e.g., corticosteroids, anakinra, or infliximab). 

Many confounding factors, such as the disease definition, the basic biochemical tests, or the treatment protocol used over the last decade, did not change. Therefore, differences in phenotype may be (partially) dependent on a difference in pathogenic mechanism. This was also suggested by our recent collaborative study, in which pre-COVID KD-patients with shock syndrome were found to have Vβ21.3+ T cell expansions similar to MIS-C [20]. While our numbers of KD cases were too small to do extensive per-year analyses, it would be interesting to further investigate in a more detailed manner if the phenotype of KD could depend on the varying infectious triggers that circulate within the population to which children are exposed. Although purely speculative, KD may indeed be a syndrome defined by clinical characteristics, which may comprise a constellation of inflammatory conditions with different etiologies but a common clinical presentation by default. 

Third, despite some clinical overlap between KD and MIS-C, important differences were found, and the incidence and phenotype of MIS-C changed over time. In comparison with KD, children with MIS-C were older, had more gastrointestinal symptoms (94.3%), and were more often admitted to the ICU [2,3]. Similar to previous literature, patients with MIS-C in our cohort also responded well to treatment with IVIG (mostly a single infusion) and corticosteroids [21,22]. Cardiac involvement differed between KD and MIS-C with MIS-C patients more often presenting with cardiac dysfunction manifested by left ventricle dysfunction, while CAAs were less frequent and usually transient [23,24,25]. In addition, immunological differences were previously identified between KD and MIS-C. While cytokine profiles overlap between these two hyperinflammatory syndromes, differences are seen both between as well as within these entities, as we showed in our previous study, and was also described by other groups [26,27,28]. Moreover, transient Vβ21.3+ T cell expansion is found in MIS-C, which normalizes during the first 10–20 days after clinical admittance [28,29]. Although this expansion is usually not found in KD patients, in our previous collaborative study, we identified four pre-COVID non-SARS-CoV-2 KD patients presenting with clinical features similar to MIS-C and Vβ21.3+ T cell expansion [20]. For one of the patients, a serum sample was available to test with VirScan, showing enrichment for antibodies against 18 human pathogens, including coronaviruses. A second patient tested positive for seasonal human coronavirus 229E by nasal PCR. Again, these findings could suggest that multiple immunological and clinical phenotypes of KD may exist, depending on the triggering pathogen involved. Additionally, among the recent SARS-CoV-2 strains, the presentation of the disease changed. Apart from the decrease in MIS-C due to the Omicron strains (Figure 2), the proportion admitted to the ICU significantly decreased over time and no more MIS-C patients were identified in The Netherlands since April 2022. This observation suggests a role for both changes in the virulence of a microbial trigger which has decreased immunoreactive properties compared to previous strains, as well as altered levels of exposure due to population-based protection against pandemic levels of spreading and herd immunity, to explain variance in KD presentations [30,31].

Several limitations surrounding our study should be noted. First, a subgroup of patients were excluded from our analyses due to uncertainty surrounding the initial diagnosis and presenting symptoms. However, inclusion of these patients in the analyses did not significantly change our results. Second, part of our patients were included retrospectively, instead of prospectively, since they were either diagnosed prior to the initiation of our Kawasaki Disease Study in 2012 or identified in peripheral hospitals. Since the retrospective and prospective KD cohorts slightly differed (Table 1), likely due to recall and/or selection bias, we excluded the retrospective KD patients (date of onset before 2012) from our longitudinal analyses. Third, the differentiation of COVID-19-triggered KD cases from MIS-C patients was challenging, but inherent to the clinical definition of MIS-C. 

## 5. Conclusions

In conclusion, KD remains a severe disease with a substantial risk of CAA development with slighter chances of normalization in older children, raising the question of whether more rigorous treatment should be initiated in these patients. Based on the phenotypical changes observed in KD and MIS-C over time, and the clinical overlap between KD and MIS-C, it could be speculated that the diagnosis of KD may coin together a spectrum of hyperinflammatory diseases with overlapping characteristics. The initial description by professor Tomisaku Kawasaki in 1974 as an acute febrile syndrome in children [1] instead of a single entity may be a foretelling denomination after all. Future studies using in-depth omics approaches should focus on distinguishing between the different disease phenotypes within this spectrum and determining the aberrant immunological mechanisms to initiate timely, optimal, and a more personalized treatment.

## Figures and Tables

**Table 1:** Baseline and clinical characteristics of the retrospective (before 2012) and prospective (after 2012) KD cohorts.

	Retrospective KD ^a^-Group <2012 (n = 562)	Prospective KD ^a^-Group ≥2012 (n = 440)	Significance (*p* Value) ^b^
Age at diagnosis (years)	2.3 (0.9–4.5)	3.1 (1.5–5.1)	<0.001
Male	340 (60.5%)	254 (57.9%)	0.4
Symptoms ^a^			
Fever ^c^	562 (100%)	440 (100%)	N/A
Rash	484 (89.5%)	354 (86.3%)	0.2
Conjunctivitis	453 (83.7%)	359 (87.3%)	0.1
Oral changes	468 (86.2%)	359 (88.4%)	0.3
Cervical lymphadenopathy	422 (79.2%)	298 (75.8%)	0.2
Changes of the extremities ^a^	451 (83.4%)	311 (77.8%)	0.04
Arthritis	68 (13.3%)	45 (10.2%)	0.2
Abdominal symptoms	N/A	N/A	N/A
Complete KD	452 (81.3%)	314 (75.5%)	0.03
Complications			
Shock ^d^	2 (0.4%)	8 (1.8%)	0.02
Respiratory failure	1 (0.2%)	3 (0.7%)	0.3
Acute kidney injury	1 (0.2%)	3 (0.7%)	0.3
Laboratory findings ^e^			
CRP, mg/L	115.0 (68.0–184.8)	125.0 (66.0–194.5)	0.8
Leukocytes, 10^9^/L	15.8 (12.0–19.9)	15.5 (11.8–19.5)	0.8
Thrombocytes, 10^9^/L	384.5 (309.3–513.0)	424.0 (308.0–552.5)	0.08
Hemoglobin, mmol/L	6.8 (6.3–7.3)	6.7 (6.2–7.4)	0.7
Albumin, g/L	32.5 (29.8–38.8)	32.0 (25.0–37.3)	0.4
Treatment			
IVIG	508 (90.9%)	404 (92.2%)	0.5
1st IVIG dose < 10 days	382 (80.6%)	296 (80.9%)	0.9
2nd IVIG dose administered	104 (21.0%)	112 (28.9%)	0.007
Corticosteroids	30 (5.5%)	61 (14.8%)	<0.001
Milrinone	1 (0.4%)	2 (0.5%)	1.0
Noradrenaline	2 (0.4%)	4 (0.9%)	0.3
ICU admission	19 (3.5%)	10 (2.5%)	0.4
Coronary artery aneurysms			
None	400 (77.5%)	330 (79.9%)	
Z score ≥ 2.5 to <5.0	52 (10.1%)	43 (10.4%)	
Z score ≥ 5 to <10	29 (5.6%)	14 (3.4%)	
Z score ≥ 10	35 (6.8%)	26 (6.3%)	0.3
Second KD-episode	2 (0.4%)	5 (1.2%)	0.1
Data are n (%) or median (interquartile range)			

^a^ American Heart Association criteria for the definition of Kawasaki disease (KD) is to have persistent fever and four of the following five mucocutaneous features: erythema and cracking of lips, strawberry tongue, and/or erythema of oral and pharyngeal mucosa; bilateral bulbar conjunctival injection without exudate; rash (maculopapular, diffuse erythroderma); erythema and edema of the hands and feet in acute phase and/or periungual desquamation in subacute phase; and cervical lymphadenopathy (>1.5 cm diameter). Incomplete KD was defined by at least two clinical criteria compatible with KD and additional laboratory or cardiac criteria. ^b^ Chi-square for categorical variables, Mann–Whitney U for continuous variables. ^c^ Fever > 38 °C. ^d^ Shock defined as needing inotropic support or fluid resuscitation > 20 mL/kg. ^e^ When multiple blood results were available, the nadir value (<2 weeks post-onset) was used in the analyses. Abbreviations: KD = Kawasaki disease, CRP = C-reactive protein, IVIG = intravenous immune globulin, and ICU = intensive care unit.

**Table 2:** (**A**) Association between the occurrence of a small, medium, or giant CAA in patients with KD and clinical characteristics using multiple logistic regression. (**B**) Association between the occurrence of giant CAAs in patients with KD and clinical characteristics using multiple logistic regression.

**(A)**			
**Clinical Characteristic**	**Odds Ratio**	**95% Confidence Interval**	***p* Value**
Male sex	2.2	1.3–3.8	0.002
Age < 1 year old	2.6	1.9–4.4	<0.001
Incomplete presentation	1.0	0.5–1.7	0.8
1st IVIG dose >10 days	2.7	1.5–4.9	0.001
IVIG resistance	2.2	1.3–3.7	0.002
CRP ≥ 188.0 mg/L	1.5	0.9–2.5	0.09
Leukocytes ≥ 16.5 × 10^9^/L	1.6	1.0–2.6	0.06
Thrombocytes ≤ 400.0 × 10^9^/L	1.5	0.9–2.5	0.1
**(B)**			
**Clinical Characteristic**	**Odds Ratio**	**95% Confidence Interval**	***p* Value**
Male sex	2.6	1.6–4.2	**<0.001**
Age < 1 year old	3.1	1.9–4.9	**0.001**
1st IVIG dose >10 days	2.8	1.7–4.9	**<0.001**
IVIG resistance	2.2	1.4–3.6	**0.001**
CRP ≥ 188.0 mg/L	1.8	1.1–2.9	0.01

Abbreviations: IVIG = intravenous immunoglobulins, and CRP = C-reactive protein.

**Table 3:** Baseline and clinical characteristics of the prospective pre-pandemic (≥2012–<2016 and ≥2016–<2020), pandemic (≥2020–<2022), and post-pandemic (≥2022–<2024) KD cohorts.

	Pre-Pandemic KD between 2012 and <2016 (n = 169)	Pre-Pandemic KD between 2016 and <2020 (n = 160)	Pandemic KD between 2020 and <2022 (n = 60)	Post-Pandemic KD between 2022 and <2024 (n = 51)	Significance (*p* Value) ^b^
Age at diagnosis (years)	2.8 (1.3–4.9)	3.2 (1.8–5.1)	3.3 (1.8–5.9)	3.2 (2.1–5.1)	0.5
Sex					
Female	57 (33.7%)	71 (44.7%)	32 (53.3%)	25 (48.0%)	
Male	112 (66.3%)	88 (55.3%)	28 (46.7%)	26 (51.0%)	0.02
Grandparents’ country of birth					
All in The Netherlands	78 (62.9%)	66 (52.0%)	17 (37.8%)	9 (28.1%)	
None in The Netherlands	24 (19.4%)	34 (26.8%)	18 (40.0%)	15 (46.9%)	0.004
Symptoms ^a^					
Fever ^c^	169 (100%)	160 (100%)	60 (100%)	51 (100%)	N/A
Rash	128 (83.1%)	132 (88.0%)	49 (86.0%)	45 (91.8%)	0.4
Conjunctivitis	135 (87.1%)	136 (90.1%)	47 (82.5%)	41 (85.4%)	0.5
Oral changes	130 (86.7%)	135 (90.0%)	51 (87.9%)	43 (89.6%)	0.8
Cervical lymphadenopathy	115 (76.2%)	117 (81.8%)	34 (63.0%)	32 (71.1%)	0.04
Changes of the extremities	110 (72.4%)	127 (87.0%)	38 (67.9%)	36 (78.3%)	0.005
Complete KD	113 (72.0%)	131 (86.2%)	36 (62.1%)	34 (69.4%)	<0.001
Complications					
Shock ^d^	2 (1.2%)	1 (0.6%)	4 (6.7%)	1 (2.0%)	0.02
Respiratory failure	1 (0.6%)	0 (0.0%)	1 (1.7%)	1 (2.0%)	0.4
Acute kidney injury	1 (0.6%)	1 (0.6%)	0 (0.0%)	1 (2.0%)	0.6
Laboratory findings ^e^					
CRP, mg/L	117.0 (54.5–171.5)	167.5 (86.8–246.3)	97.0 (67.0–169.0)	103.0 (53.3–170.3)	0.003
Leukocytes, 10^9^/L	15.1 (11.5–19.0)	16.7 (11.7–20.3)	14.3 (11.8–18.4)	15.9 (13.3–19.6)	0.4
Thrombocytes, 10^9^/L	401.0 (293.0–493.0)	435.0 (309.5–574.5)	472.0 (326.0–587.0)	455.0 (355.8–639.0)	0.04
Hemoglobin, mmol/L	7.1 (6.5–7.6)	6.6 (6.2–7.4)	6.4 (6.0–7.2)	6.5 (5.8–7.0)	<0.001
Albumin, g/L	36.0 (26.0–40.0)	32.0 (24.8–38.0)	32.0 (27.0–37.0)	28.5 (24.0–36.5)	0.3
Treatment					
IVIG	157 (92.9%)	147 (91.9%)	52 (88.1%)	48 (96.0%)	0.5
1st IVIG dose <10 days	112 (81.2%)	113 (83.1%)	39 (79.6%)	32 (74.4%)	0.7
2nd IVIG dose administered	44 (29.5%)	47 (33.3%)	12 (23.1%)	9 (20.0%)	0.3
Corticosteroids	16 (10.4%)	22 (14.6%)	14 (23.7%)	9 (19.1%)	0.08
ICU admission	4 (2.7%)	2 (1.4%)	3 (5.1%)	1 (2.1%)	0.4
Coronary artery aneurysms					
None	124 (78.0%)	124 (81.0%)	46 (86.8%)	34 (73.9%)	
Z score ≥ 2.5 to <5.0	20 (12.6%)	13 (8.5%)	2 (3.8%)	8 (17.4%)	
Z score ≥ 5 to <10	3 (1.9%)	6 (3.9%)	4 (7.5%)	1 (2.2%)	
Z score ≥ 10	12 (7.5%)	10 (6.6%)	1 (1.9%)	3 (6.5%)	N/A
Second KD-episode	2 (1.2%)	3 (2.2%)	0 (0%)	0 (0%)	0.7
Data are n (%) or median (interquartile range)					

^a^ American Heart Association criteria for the definition of Kawasaki disease (KD) is to have persistent fever and four of the following five mucocutaneous features: erythema and cracking of lips, strawberry tongue, and/or erythema of oral and pharyngeal mucosa; bilateral bulbar conjunctival injection without exudate; rash (maculopapular, diffuse erythroderma); erythema and edema of the hands and feet in acute phase and/or periungual desquamation in subacute phase; and cervical lymphadenopathy (>1.5 cm diameter). Incomplete KD was defined by at least two clinical criteria compatible with KD and additional laboratory or cardiac criteria. ^b^ Fisher–Freeman–Halton exact test for categorical variables, and Kruskal–Wallis adjusted by the Bonferroni correction for multiple tests for continuous variables. ^c^ Fever > 38 °C. ^d^ Shock defined as needing inotropic support or fluid resuscitation > 20 mL/kg. ^e^ When multiple blood results were available, the nadir value (<2 weeks post-onset) was used in the analyses. Abbreviations: KD = Kawasaki disease, CRP = C-reactive protein, IVIG = intravenous immune globulin, and ICU = intensive care unit.

**Table 4:** Over-time changes per year in KD phenotype from 2020 to 2023 (prospectively included patients).

	Diagnosis in 2020 (n = 28)	Diagnosis in 2021 (n = 25)	Diagnosis in 2022 (n = 30)	Diagnosis in 2023 (n = 27)	Significance (*p* Value) ^b^
Age at diagnosis (years)	4.3 (2.1–8.3)	2.9 (1.2–4.0)	3.5 (2.7–5.5)	2.5 (0.7–3.9)	0.05
Sex					
Female	14 (50%)	13 (52.0%)	15 (50%)	15 (55.6%)	
Male	14 (50%)	12 (48.0%)	15 (50%)	12 (44.4%)	0.9
Grandparents’ country of birth					
All in The Netherlands	7 (38.9%)	6 (28.6%)	9 (47.4%)	4 (21.1%)	
None in The Netherlands	6 (33.3%)	11 (52.4%)	7 (36.8%)	9 (47.4%)	0.6
Symptoms ^a^					
Fever ^c^	28 (100%)	25 (100%)	30 (100%)	27 (100%)	N/A
Rash	26 (92.9%)	17 (73.9%)	26 (96.3%)	24 (88.9%)	0.1
Conjunctivitis	23 (82.1%)	20 (87.0%)	22 (81.5%)	22 (84.6%)	1.0
Oral changes	24 (88.9%)	20 (83.3%)	26 (92.9%)	23 (88.5%)	0.7
Cervical lymphadenopathy	13 (50%)	17 (77.3%)	17 (65.4%)	19 (79.2%)	0.1
Changes of the extremities	20 (74.1%)	14 (60.9%)	19 (73.1%)	21 (84.0%)	0.4
Complete KD	18 (64.3%)	14 (60.9%)	19 (67.9%)	19 (70.4%)	0.9
Complications					
Shock ^d^	2 (7.1%)	1 (4.0%)	2 (6.7%)	0 (0%)	0.7
Respiratory failure	1 (3.6%)	0 (0%)	1 (3.3%)	0 (0%)	1.0
Acute kidney injury	0 (0%)	0 (0%)	1 (3.3%)	0 (0%)	1.0
Treatment					
IVIG	22 (81.5%)	23 (92.0%)	29 (96.7%)	25 (96.2%)	0.2
1st IVIG dose <10 days	18 (81.8%)	16 (76.2%)	20 (76.9%)	16 (72.7%)	0.9
2nd IVIG dose administered	7 (31.8%)	4 (17.4%)	3 (11.1%)	7 (29.2%)	0.2
Corticosteroids	8 (29.6%)	4 (16.0%)	6 (21.4%)	4 (16.0%)	0.6
ICU admission	3 (11.1%)	0 (0%)	1 (3.5%)	0 (0%)	0.1
Coronary artery aneurysms					
None	21 (87.5%)	19 (86.5%)	24 (80.0%)	18 (75.0%)	
Z score ≥ 2.5 to <5.0	0 (0%)	1 (4.5%%)	3 (10.0%)	5 (20.8%)	
Z score ≥ 5 to <10	3 (12.5%)	1 (4.5%)	1 (3.3%)	0 (0%)	
Z score ≥ 10	0 (0%)	1 (4.5%)	2 (6.7%)	1 (4.2%)	0.1
Second KD-episode	0 (0%)	0 (0%)	0 (0%)	0 (0%)	N/A
Data are n (%) or median (interquartile range)					

^a^ American Heart Association criteria for the definition of Kawasaki disease (KD) is to have persistent fever and four of the following five mucocutaneous features: erythema and cracking of lips, strawberry tongue, and/or erythema of oral and pharyngeal mucosa; bilateral bulbar conjunctival injection without exudate; rash (maculopapular, diffuse erythroderma); erythema and edema of the hands and feet in acute phase and/or periungual desquamation in subacute phase; and cervical lymphadenopathy (>1.5 cm diameter). Incomplete KD was defined by at least two clinical criteria compatible with KD and additional laboratory or cardiac criteria. ^b^ Fisher–Freeman–Halton exact test for categorical variables, and Kruskal–Wallis adjusted by the Bonferroni correction for multiple tests for continuous variables. ^c^ Fever > 38 °C. ^d^ Shock defined as needing inotropic support or fluid resuscitation > 20 mL/kg. Abbreviations: KD = Kawasaki disease, CRP = C-reactive protein, IVIG = intravenous immune globulin, and ICU = intensive care unit.

**Table 5:** Baseline characteristics and clinical phenotype of prospectively included MIS-C and KD patients included within the MIS-C pandemic period (2020 to April 2022).

	Pandemic KD ^a^ between 2020 and <2022 (n = 60)	MIS-C ^b^ (n = 73)	Significance Pandemic KD vs. MIS-C (*p* Value) ^c^
Age at diagnosis (years)	3.3 (1.8–5.9)	11.3 (6.0–13.8)	<0.001
Male	28 (46.7%)	48 (65.8%)	0.02
Grandparents’ country of birth			
All in The Netherlands	17 (37.8%)	28 (44.4%)	
None in The Netherlands	18 (40.0%)	25 (39.7%)	0.6
Symptoms ^a^			
Fever ^d^	60 (100%)	73 (100%)	N/A
Rash	49 (86.0%)	43 (60.6%)	0.002
Conjunctivitis	47 (82.5%)	52 (73.2%)	0.3
Oral changes	51 (87.9%)	47 (66.2%)	0.006
Cervical lymphadenopathy	34 (63.0%)	34 (50.0%)	0.2
Changes of the extremities ^a^	38 (67.9%)	25 (36.8%)	<0.001
Arthritis	4 (13.3%)	7 (9.6%)	1.0
Abdominal symptoms	N/A	66 (94.3%)	N/A
Complete KD	36 (62.1%)	22 (31.0%)	<0.001
Complications			
Shock ^e^	4 (6.7%)	33 (45.2%)	<0.001
Respiratory failure	1 (1.7%)	19 (26.0%)	<0.001
Acute kidney injury	0 (0.0%)	14 (19.2%)	<0.001
Laboratory findings ^f^			
CRP, mg/L	97.0 (67.0–169.0)	188.0 (129.0–283.0)	<0.001
Leukocytes, 10^9^/L	14.3 (11.8–18.4)	14.1 (9.8–21.2)	0.9
Thrombocytes, 10^9^/L	472.0 (326.0–587.0)	350.0 (203.0–523.5)	0.02
Hemoglobin, mmol/L	6.4 (6.0–7.2)	6.6 (5.9–7.2)	0.9
Albumin, g/L	32.0 (27.0–37.0)	30.0 (26.0–36.0)	0.7
Treatment			
IVIG	52 (88.1%)	66 (91.7%)	0.6
1st IVIG dose <10 days	39 (79.6%)	62 (95.4%)	0.02
2nd IVIG dose administered	12 (23.1%)	17 (25.8%)	0.8
Corticosteroids	14 (23.7%)	55 (75.3%)	<0.001
Milrinone	1 (1.7%)	17 (23.3%)	<0.001
Noradrenaline	1 (1.7%)	17 (23.3%)	<0.001
ICU admission	3 (5.1%)	28 (38.4%)	<0.001
Coronary artery aneurysms			
None	46 (86.8%)	65 (90.3%)	
Z score ≥ 2.5 to <5.0	2 (3.8%)	6 (8.3%)	
Z score ≥ 5 to <10	4 (7.5%)	1 (1.4%)	
Z score ≥ 10	1 (1.9%)	0 (0%)	0.01
Cardiac dysfunction ^g^	0 (0.0%)	39 (81.3%)	N/A
Second KD-episode	0 (0.0%)	0 (0%)	N/A
Data are n (%) or median (interquartile range)			

^a^ American Heart Association criteria for the definition of Kawasaki disease (KD) is to have persistent fever and four of the following five mucocutaneous features: erythema and cracking of lips, strawberry tongue, and/or erythema of oral and pharyngeal mucosa; bilateral bulbar conjunctival injection without exudate; rash (maculopapular, diffuse erythroderma); erythema and edema of the hands and feet in acute phase and/or periungual desquamation in subacute phase; and cervical lymphadenopathy (>1.5 cm diameter). Incomplete KD was defined by at least two clinical criteria compatible with KD and additional laboratory or cardiac criteria. ^b^ MIS-C according to criteria by Centers for Disease Control and Prevention (CDC) and World Health Organization (WHO). ^c^ Fisher–Freeman–Halton exact test for categorical variables, Mann–Whitney U for continuous variables. ^d^ Fever > 38 °C. ^e^ Shock defined as needing inotropic support or fluid resuscitation > 20 mL/kg. ^f^ When multiple blood results were available, the nadir value (<2 weeks post-onset) was used in the analyses. ^g^ Cardiac dysfunction was defined as a left ventricle ejection fraction < 50% and/or a fractional shortening < 28%. Abbreviations: KD = Kawasaki disease, MIS-C = multisystem inflammatory syndrome in children, CRP = C-reactive protein, IVIG = intravenous immune globulin, and ICU = intensive care unit.

**Table 6:** Over-time changes in MIS-C phenotype in 2020, 2021, and 2022 (prospectively included patients).

	Diagnosis in 2020 (n = 21)	Diagnosis in 2021 (n = 29)	Diagnosis in 2022 (n = 23)	Significance (*p* Value) ^b^
Age at diagnosis (years)	11.8 (6.9–16.3)	11.3 (5.4–13.0)	10.8 (6.3–12.7)	0.4
Sex				
Female	9 (42.9%)	11 (37.9%)	5 (21.7%)	
Male	12 (57.1%)	18 (62.1%)	18 (78.3%)	0.3
Grandparents’ country of birth				
All in The Netherlands	6 (31.6%)	8 (36.4%)	14 (63.6%)	
None in The Netherlands	10 (52.6%)	8 (36.4%)	7 (31.8%)	0.1
Symptoms ^a^				
Fever ^c^	20 (100%)	29 (100%)	22 (100%)	N/A
Rash	14 (66.7%)	15 (51.7%)	14 (66.7%)	0.4
Conjunctivitis	13 (61.9%)	22 (75.9%)	17 (81.0%)	0.3
Oral changes	12 (57.1%)	20 (69.0%)	15 (71.4%)	0.7
Cervical lymphadenopathy	14 (66.7%)	11 (39.3%)	9 (47.4%)	0.2
Changes of the extremities	8 (38.1%)	14 (50.0%)	3 (15.8%)	0.05
Abdominal symptoms	20 (95.2%)	28 (96.6%)	18 (90.0%)	0.6
Complete KD	9 (42.9%)	8 (27.6%)	5 (23.8%)	0.5
Complications				
Shock ^d^	9 (42.9%)	17 (58.6%)	7 (30.4%)	0.1
Respiratory failure	9 (42.9%)	9 (31.0%)	1 (4.3%)	0.007
Acute kidney injury	6 (28.6%)	8 (27.6%)	0 (0.0%)	0.009
Laboratory findings ^e^				
CRP, mg/L	254.5 (117.0–323.3)	212.0 (129.0–264.0)	154.0 (124.5–196.0)	0.2
Leukocytes, 10^9^/L	16.0 (9.6–22.9)	16.3 (10.1–22.6)	13.2 (9.1–14.9)	0.08
Thrombocytes, 10^9^/L	473.0 (214.5–639.5)	404.0 (239.5–552.5)	277.5 (150.3–371.0)	0.1
Hemoglobin, mmol/L	6.5 (5.6–7.3)	6.3 (5.9–6.9)	6.9 (6.4–7.3)	0.2
Albumin, g/L	30.0 (25.0–35.0)	27.0 (24.5–30.0)	39.0 (34.0–41.0)	<0.001
NT-pro-BNP, ng/L	4414.5 (1975.0–12,364.0)	4301.0 (1682.0–15,710.0)	2238.0 (697.5–5763.5)	0.1
Troponin T, ng/L	46.0 (12.0–65.0)	48.5 (15.0–120.0)	35.0 (10.5–106.8)	0.7
Treatment				
IVIG	18 (85.7%)	27 (93.1%)	21 (95.5%)	0.6
1st IVIG dose <10 days	17 (94.4%)	26 (96.3%)	19 (95.0%)	1.0
2nd IVIG dose administered	6 (33.3%)	6 (22.2%)	5 (23.8%)	0.7
Corticosteroids	11 (52.4%)	26 (89.7%)	18 (78.3%)	0.01
Milrinone	7 (33.3%)	10 (34.5%)	0 (0.0%)	0.006
Noradrenaline	8 (38.1%)	12 (41.4%)	0 (0.0%)	0.002
ICU admission	11 (52.4%)	13 (44.8%)	4 (17.4%)	0.04
Coronary artery aneurysms				
None	15 (75.0%)	27 (93.1%)	23 (100%)	
Z score ≥ 2.5 to <5.0	4 (20.0%)	2 (6.9%)	0 (0.0%)	
Z score ≥ 5 to <10	1 (5.0%)	0 (0.0%)	0 (0.0%)	
Z score ≥ 10	0 (0.0%)	0 (0.0%)	0 (0.0%)	0.02
Second KD-episode	0 (0.0%)	0 (0.0%)	0 (0.0%)	N/A
Data are n (%) or median (interquartile range)				

^a^ American Heart Association criteria for the definition of Kawasaki disease (KD) is to have persistent fever and four of the following five mucocutaneous features: erythema and cracking of lips, strawberry tongue, and/or erythema of oral and pharyngeal mucosa; bilateral bulbar conjunctival injection without exudate; rash (maculopapular, diffuse erythroderma); erythema and edema of the hands and feet in acute phase and/or periungual desquamation in subacute phase; and cervical lymphadenopathy (>1.5 cm diameter). Incomplete KD was defined by at least two clinical criteria compatible with KD and additional laboratory or cardiac criteria. ^b^ Fisher–Freeman–Halton exact test for categorical variables, and Kruskal–Wallis for continuous variables. ^c^ Fever > 38 °C. ^d^ Shock defined as needing inotropic support or fluid resuscitation > 20 mL/kg. ^e^ When multiple blood results were available, the nadir value (<2 weeks post-onset) was used in the analyses. Abbreviations: KD = Kawasaki disease, CRP = C-reactive protein, IVIG = intravenous immune globulin, and ICU = intensive care unit.

## Data Availability

Data is contained within the article.
